# Therapierefraktäre Anämie bei einem 35-jährigen Dialysepatienten nach Herztransplantation

**DOI:** 10.1007/s00108-021-00955-9

**Published:** 2021-02-12

**Authors:** Christoph Schröder, Johannes Roeles, Adrian Schwarzer, Michael Heuser, Jennifer Retzlaff, Marcus Hiß

**Affiliations:** 1 Klinik für Nieren- und Hochdruckerkrankungen, Medizinische Hochschule Hannover, Carl-Neuberg-Straße 1, 30625 Hannover, Deutschland; 2 Klinik für Hämatologie, Hämostaseologie, Onkologie und Stammzelltransplantation, Medizinische Hochschule Hannover, Hannover, Deutschland

**Keywords:** Parvovirus B19, Isolierte aplastische Anämie, Riesenproerythroblasten, Intravenöse Immunglobuline, Polymerase-Kettenreaktion, Parvovirus B19, human, Red cell aplasia, pure, Megaloblasts, Immunoglobulins, intravenous, Polymerase chain reaction

## Abstract

Es wird über einen 35-jährigen Dialysepatienten nach Herztransplantation berichtet, der eine „pure red cell aplasia“ aufwies. Serologisch fand sich ein positiver Parvovirus-B19-Immunglobulin-M-Titer, im Direktnachweis mittels Polymerase-Kettenreaktion zeigten sich 80 Mrd. IU/ml. In der zytologischen Untersuchung des Knochenmarks gelang der Nachweis von Riesenproerythroblasten, pathognomonisch für eine Parvovirus-B19-Infektion. Zur Therapie erfolgte die hoch dosierte Gabe von Immunglobulinen über 5 Tage. Bei Wiedervorstellung nach vier Wochen zeigte sich die Anämie deutlich gebessert. Bei Patienten nach Organtransplantation mit hyporegenerativer Anämie sollte eine Parvovirus-B19-Infektion stets ausgeschlossen werden.

## Abstract (en)

This article presents a case of pure red cell aplasia in a 35-year-old heart transplant recipient on chronic hemodialysis. Elevated parvovirus B19 immunoglobulin M blood levels were detected along with a high viral load of 80 billion IU/ml quantified by polymerase chain reaction. Bone marrow examination revealed giant proerythroblasts confirming parvovirus B19 infection. High-dose intravenous immunoglobulin was used for treatment. Anaemia had significantly improved 4 weeks later. Parvovirus B19 infection should be excluded in organ transplant recipients with anaemia due to ineffective erythropoiesis.

## Anamnese

Ein 35-jähriger Patient mit seit zwei Jahren bestehender, dialysepflichtiger Niereninsuffizienz nach orthotoper Herztransplantation bei postmyokarditischer Kardiomyopathie wurde uns aufgrund einer therapierefraktären Anämie zugewiesen.

Seit 6 Monaten bestand eine erythropoetinresistente, hyporegenerative Anämie, die mit regelmäßigen Transfusionen behandelt wurde. Im Vorfeld der stationären Aufnahme war eine Blutungsquelle mittels Gastro- und Koloskopie sowie Kapselendoskopie ausgeschlossen worden. Es bestand kein Eisenmangel, ebenso kein Mangel an Vitamin B12 oder Folsäure. Auch Antikörper gegen Erythropoetin konnten nicht nachgewiesen werden. Eine vorangegangene Knochenmarkbiopsie hatte keinen Hinweis auf ein myelodysplastisches Syndrom oder eine monoklonale Plasmazellvermehrung ergeben, zeigte jedoch eine auf 2 % der Norm reduzierte Erythropoese. Eine zytologische Beurteilung entfiel bei Punctio sicca.

Seit Krankheitsbeginn hatte der Patient insgesamt 8 kg Körpergewicht verloren, sodass auch eine maligne Grunderkrankung denkbar war. Fieber oder eine Nachtschweißsymptomatik bestand jedoch zu keinem Zeitpunkt. Aufgrund des stark reduzierten Allgemeinzustands war der Patient nur noch am Rollator mobil. Er ist Vater von drei gesunden Kindern im schulpflichtigen Alter. Das immunsuppressive Regime bestand aus Everolimus, Tacrolimus und Prednisolon p.o.

## Klinischer Befund

Der Patient präsentierte sich dyspnoisch mit blassem Hautkolorit. Das Körpergewicht lag bei 80 kg bei einer Körpergröße von 185 cm (Body-Mass-Index 23,7 kg/m^2^). Der Blutdruck war mit 108/61 mm Hg erniedrigt, die Herzfrequenz mit 103/min erhöht. Es bestand kein Fieber (37,1 °C), die pulsoxymetrisch gemessene Sauerstoffsättigung betrug 100 %. Das Integument zeigte sich unauffällig, es bestand eine Euvolämie.

## Diagnostik

Im *Aufnahmelabor* zeigte sich eine normochrome, hyporegenerative Anämie (Hämoglobin 7,6 g/dl) mit einer stark erniedrigten Retikulozytenzahl (2/nl; Norm 25–105/nl; Retikulozytenproduktionsindex 0). Thrombozyten und Leukozyten waren normwertig, im *Differenzialblutbild* zeigte sich eine isolierte Lymphozytopenie. Darüber hinaus bestand eine Eisenüberladung (Ferritin 800 ng/ml, Transferrinsättigung 50 %), ein Vitamin-B12- oder Folsäuremangel konnte ausgeschlossen werden. Das C‑reaktive Protein war mit 7,3 mg/l nur leicht erhöht. *Serologisch* fand sich ein positiver Parvovirus-B19-Immunglobulin-M(IgM)-Titer, bei negativem Parvovirus-B19-Immunglobulin G (IgG). Aktive Infektionen durch das Zytomegalievirus und Epstein-Barr-Virus wurden ausgeschlossen. Eine *Abdomensonographie* erbrachte einen Normalbefund.

## Differenzialdiagnosen

-  Differenzialdiagnostisch wurden zu diesem Zeitpunkt eine medikamentös-toxische Knochenmarkschädigung (insbesondere mTOR-Inhibitor-vermittelt) und eine infektiologische Ursache (vor allem eine Virusinfektion) in Betracht gezogen.

## Weiterer Verlauf

**Figure 1:**
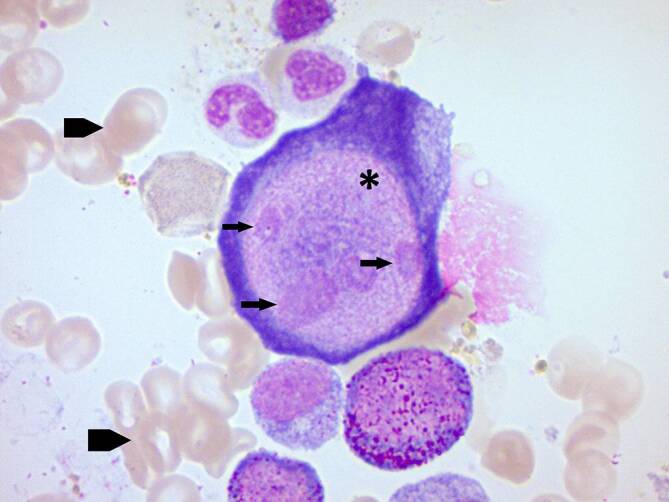

Die Everolimusbehandlung wurde pausiert, ein Hydrokortisonperfusor etabliert. Die *Knochenmarkaspiration* wurde wiederholt. In der *zytologischen Untersuchung des Knochenmarks* zeigte sich ein hypozelluläres Mark mit einer hochgradigen Reduktion der ausreifenden Erythropoese bei normaler Granulo- und Megakaryopoese. Auffällig war der Nachweis von Riesenproerythroblasten (Gigantoblasten) mit nukleären Viruseinschlüssen (Abb. 1). Zusätzlich konnte mittels *Polymerase-Kettenreaktion* (PCR) eine Parvoviruslast von 80 Mrd. IU/ml im Serum und 50 Mrd. IU/ml im Knochenmark gesichert werden. Im weiteren stationären Verlauf aggravierte die Anämiesymptomatik unter einem Hämoglobinwert von 6,4 g/dl.

## Diagnose

- „Pure red cell aplasia“ (PRCA) bei Parvovirus-B19-Infektion

## Therapie und Verlauf

Eine hoch dosierte Immunglobulintherapie mit 2 g/kg über 5 Tage wurde initiiert. Zum Zeitpunkt der Entlassung erfolgte die Reduktion der Everolimus- und Tacrolimusdosis unter vorübergehender Erhöhung des oralen Prednisolons.

Vier Wochen später stellte sich der Patient in gutem Allgemeinzustand vor. Der Hämoglobinwert zeigte sich mit 10,8 g/dl gebessert, ohne zwischenzeitlichen Transfusionsbedarf. Die Parvovirus-B19-Viruslast im Serum war mit 3000 IU/ml rückläufig, eine Parvovirus-B19-IgM-zu-IgG-Serokonversion hatte stattgefunden.

## Diskussion

### Krankheitsbild der Parvovirus-B19-Infektion

Eine Infektion mit dem Parvovirus B19 verläuft bei immunkompetenten Patienten in der Regel asymptomatisch. Die häufigste klinische Manifestation ist im Kindesalter das Erythema infectiosum. Auf ein unspezifisches Prodromalstadium mit Fieber, Schüttelfrost, Zephalgien und Myalgien folgen ein schmetterlingsförmiges Gesichtserythem („slapped cheek“) und ein girlandenförmiges Ganzkörperexanthem [10].

Nach Organtransplantation ist das Risiko der Chronifizierung einer Parvovirus-B19-Infektion erhöht

Bei Patienten mit chronischen Vorerkrankungen zeigt sich jedoch oft ein hiervon abweichender klinischer Verlauf [9]. Insbesondere Patienten nach Organtransplantation haben ein erhöhtes Risiko für eine Chronifizierung der Infektion. Die weitaus häufigste Manifestation innerhalb dieses Patientenkollektivs ist eine progrediente Anämie mit körperlicher Schwäche, Dyspnoe und orthostatischer Dysregulation [2, 5]. Ein Ganzkörperexanthem tritt in nur 13 % der Fälle auf. Etwas seltener zeigen sich auch andere Organmanifestationen wie eine Myokarditis, eine Pneumonie oder Hepatitis. Zu einer Transplantatdysfunktion oder zum Transplantatverlust kommt es in etwa 10 % der Fälle. Hier ist die Dysfunktion des Nierentransplantats mit 15,6 % führend, gefolgt von Herz- und Lungentransplantaten mit 8,3 % [5].

Die Virusübertragung erfolgt mittels Tröpfcheninfektion. Auf eine Virusreplikation im Nasopharyngealraum folgt etwa eine Woche später eine Virämie [1]. Die Virusreplikation in erythropoetischen Progenitorzellen führt dann zur Herunterregulation von Erythropoetinrezeptoren, zum Zellzyklusarrest und letztlich zur Apoptose der Zelle [6].

### Differenzialdiagnose bei anämischen Patienten nach Organtransplantation

Generell sollten bei Patienten nach Organtransplantation, die eine Anämie aufweisen, neben unspezifischen Ursachen wie einem Mangel an Eisen, Vitamin B12 oder Folsäure auch transplantationsspezifische Ursachen ausgeschlossen werden. Hierzu gehören unter anderem medikamentös-toxische Knochenmarkschädigungen. Diese können zum einen durch immunsuppressive Substanzen wie Azathioprin, Mycophenolatmofetil oder mTOR-Inhibitoren bedingt sein, aber auch durch antimikrobielle Prophylaxen, beispielsweise mit Trimethoprim [8].

Des Weiteren sind chronische Infektionen in Erwägung zu ziehen. Insbesondere virale Erreger spielen eine wichtige Rolle. Neben dem Parvovirus B19 ist an eine Infektion mit dem Zytomegalievirus oder dem Epstein-Barr-Virus zu denken, seltener auch an das Varizella-Zoster-Virus, das humane Herpesvirus 6 und 8 sowie an das BK-Virus. Auch nichtinfektiöse Erkrankungen wie das Posttransplantationslymphom oder eine thrombotische Mikroangiopathie sollten differenzialdiagnostisch in Erwägung gezogen werden [8].

### Diagnostik der Parvovirus-B19-Infektion

Zum Ausschluss einer Parvovirus-B19-Infektion sollte die serologische Initialdiagnostik um eine PCR ergänzt werden [4], da die antikörpervermittelte Immunantwort inadäquat oder verzögert verlaufen kann. In einem Kollektiv von 98 Patienten mit Parvovirus-B19-Infektion nach Transplantation zeigten 29 % initial eine negative IgM-Serologie [5]. Der Virusdirektnachweis aus infiziertem Gewebe, beispielsweise der Niere, Leber, Lunge oder des Knochenmarks, kann bei negativem serologischem Nachweis und fortbestehendem Verdacht sinnvoll sein. In der zytologischen Untersuchung des Knochenmarks zeigt sich eine hochgradige Reduktion bis hin zum völligen Fehlen von Vorstufen der Erythropoese. Häufig lassen sich charakteristische Riesenproerythroblasten mit eosinophilen nukleären Einschlüssen nachweisen [7].

### Therapie

Eine spezifische antivirale Therapie existiert derzeit nicht. Therapeutisch kommt die intravenöse Gabe von Immunglobulinen (IVIG) in hoher Dosierung zum Einsatz. Die American Society of Transplantation empfiehlt eine Dosierung von 400 mg/kg täglich. Falls möglich sollte eine Reduktion der Immunsuppression erfolgen [4]. Als Nebenwirkungen können Fieber, Schüttelfrost, Zephalgien, Myalgien, Übelkeit, Blutdruckentgleisungen, Angina pectoris und eine akute Nierenfunktionsverschlechterung auftreten. Die Verabreichung der Gesamtdosis über 5 Tage im Vergleich zu einem 2‑tägigen Therapieregime erhöht die IVIG-Verträglichkeit [3].

Therapeutisch kommt die intravenöse Gabe von Immunglobulinen in hoher Dosierung zum Einsatz

Die Ansprechrate der PRCA auf die IVIG-Behandlung liegt bei 93 % [3]. Jedoch gibt es zurzeit keine prospektiven, kontrollierten Untersuchungen, die den Effekt dieser Behandlung beweisen. In einzelnen kleinen Kohorten von Patienten mit PRCA bei Parvovirus-B19-Infektion nach Transplantation beträgt die spontane Remissionsrate etwa 60 % [2, 5]. Die Rezidivrate der Parvovirus-B19-assoziierten PRCA liegt bei 28–34 %, unabhängig von der Immunglobulingesamtdosis (≤2 g/kg oder > 2 g/kg; [3, 5]). Die American Society of Transplantation empfiehlt in diesen Fällen weitere IVIG-Zyklen in 4‑wöchigen Abständen bis zum Sistieren der Symptomatik [4].

Der Stellenwert der PCR in der Verlaufskontrolle ist nicht gesichert. In seltenen Fällen kann die Virus-DNA bis zu 3 Jahre nach dem akuten Infekt nachgewiesen werden. Eine solche „DNAämie“ sollte lediglich zur Veranlassung einer regelmäßigen Hämoglobinmessung führen, beim Auftreten einer erneuten Anämie sollte eine IVIG-Behandlung erfolgen [4].

## Fazit für die Praxis

- Eine Parvovirusinfektion manifestiert sich bei Patienten nach Organtransplantation am häufigsten als Anämie.
- Die initiale serologische Diagnostik sollte in diesem Patientenkollektiv um eine Polymerase-Kettenreaktion (PCR) ergänzt werden.
- Therapeutisch kommen Immunglobuline in einer Dosierung von 400 mg/kg täglich über 5 Tage zum Einsatz, falls möglich sollte eine Reduktion der Immunsuppression erfolgen.
- Das Therapiemonitoring sollte in Bezug auf die klinische Symptomatik erfolgen, der Stellenwert der PCR in der Verlaufskontrolle ist nicht gesichert.
